# Monitoring the initial pulmonary absorption of two different beclomethasone dipropionate aerosols employing a human lung reperfusion model

**DOI:** 10.1186/1465-9921-6-21

**Published:** 2005-02-24

**Authors:** Matthias Freiwald, Anagnostis Valotis, Andreas Kirschbaum, Monika McClellan, Thomas Mürdter, Peter Fritz, Godehard Friedel, Michael Thomas, Petra Högger

**Affiliations:** 1 Institut für Pharmazie und Lebensmittelchemie, Bayerische Julius-Maximilians-Universität, Würzburg, Germany; 2 Klinik Schillerhöhe der LVA Württemberg, Gerlingen, Germany; 3 Dr. Margarete Fischer-Bosch-Institut für Klinische Pharmakologie, Stuttgart, Germany; 4 Pathologisches Institut am Robert Bosch Krankenhaus, Stuttgart, Germany; 5 Internistische Onkologie der Thoraxtumoren, Thoraxklinik GmbH am Universitätsklinikum Heidelberg, Heidelberg, Germany

## Abstract

**Background:**

The pulmonary residence time of inhaled glucocorticoids as well as their rate and extend of absorption into systemic circulation are important facets of their efficacy-safety profile. We evaluated a novel approach to elucidate the pulmonary absorption of an inhaled glucocorticoid. Our objective was to monitor and compare the combined process of drug particle dissolution, pro-drug activation and time course of initial distribution from human lung tissue into plasma for two different glucocorticoid formulations.

**Methods:**

We chose beclomethasone dipropionate (BDP) delivered by two different commercially available HFA-propelled metered dose inhalers (Sanasthmax^®^/Becloforte™ and Ventolair^®^/Qvar™). Initially we developed a simple dialysis model to assess the transfer of BDP and its active metabolite from human lung homogenate into human plasma. In a novel experimental setting we then administered the aerosols into the bronchus of an extracorporally ventilated and reperfused human lung lobe and monitored the concentrations of BDP and its metabolites in the reperfusion fluid.

**Results:**

Unexpectedly, we observed differences between the two aerosol formulations Sanasthmax^®^/Becloforte™ and Ventolair^®^/Qvar™ in both the dialysis as well as in the human reperfusion model. The HFA-BDP formulated as Ventolair^®^/Qvar™ displayed a more rapid release from lung tissue compared to Sanasthmax^®^/Becloforte™. We succeeded to explain and illustrate the observed differences between the two aerosols with their unique particle topology and divergent dissolution behaviour in human bronchial fluid.

**Conclusion:**

We conclude that though the ultrafine particles of Ventolair^®^/Qvar™ are beneficial for high lung deposition, they also yield a less desired more rapid systemic drug delivery. While the differences between Sanasthmax^®^/Becloforte™ and Ventolair^®^/Qvar™ were obvious in both the dialysis and lung perfusion experiments, the latter allowed to record time courses of pro-drug activation and distribution that were more consistent with results of comparable clinical trials. Thus, the extracorporally reperfused and ventilated human lung is a highly valuable physiological model to explore the lung pharmacokinetics of inhaled drugs.

## Introduction

Current asthma management guidelines recommend inhaled glucocorticoids as preferred therapy for control of mild persistent, moderate and severe asthma [1,2]. Glucocorticoids are the most effective anti-inflammatory agents and inhalation is an efficient way to deposit the compound in the therapeutic target tissue. The dose and the percentage of lung deposition as well as the specific receptor binding affinity determine the therapeutic efficacy of the corticosteroid [3,4]. Prolonged residence of an inhaled glucocorticoid in the lung tissue is associated with an extended duration of action. In contrast, the rate and extend of absorption of the glucocorticoid into systemic circulation might result in systemic adverse effects such as adrenal suppression or decreased bone mineral density [5]. Though the tissue residence time and the time course of distribution into systemic circulation significantly contribute to the risk-benefit value of inhaled corticosteroids [4,6] the precise determination of the pulmonary absorption is a challenge.

The time course of inhaled drug absorption has been frequently studied in life animals [7]. For this purpose, the drugs are usually administered intratracheally as solution or via intratracheal nebulization [8]. This, however, is not directly comparable to the administration of therapeutically used glucocorticoids in humans which are usually formulated as aerosols or dry powder inhalers containing micronized drug crystals.

The *in vivo *distribution of inhaled glucocorticoids between human lung tissue and plasma has been determined in patients undergoing thoracotomy. During surgery tissue and plasma samples were obtained and analyzed for drug concentrations which were found to be significantly higher in lung tissue compared to plasma [9-11]. The strength of this type of evaluation is that tissue concentrations of the drug can be measured up to ten or more hours after inhalation. However, one patient provides one data point only and thus many patients are needed to sufficiently describe a time course of tissue – plasma distribution.

Plasma concentrations of glucocorticoids after inhalation of therapeutic doses are low and thus highly sensitive analytical methods are required [12]. Blood samples from an antecubital vein are collected at defined time intervals after inhalation and analyzed. Since an unknown percentage of the corticosteroid in this blood sample might have already undergone metabolization when passing the liver, it cannot be excluded that the measured concentration underestimates the amount of active drug delivered from the lung tissue.

The purpose of this study was to monitor and compare the combined process of drug particle dissolution, pro-drug activation and time course of distribution from human lung tissue into plasma in the absence of hepatic metabolism. We chose beclomethasone dipropionate (BDP) as a model compound because different formulations are commercially available and pharmacokinetic data from clinical studies is accessible for comparison. BDP is activated in human lung tissue to yield its active metabolite beclomethasone-17-monopropionate (17-BMP) [13]. We initially developed a dialysis model to monitor the drug transfer of BDP and metabolites from human lung tissue homogenate into human plasma. To compare the results of these experiments with a more physiological model we studied drug diffusion kinetics employing resected intact human lung lobes. This human lung reperfusion model was previously developed by Linder et al. [14] and successfully used to study the uptake kinetics of anticancer agents from the perfusion fluid into normal and tumour lung tissue [15,16]. To our knowledge, human lung reperfusion settings have not been employed so far for evaluation of distribution kinetics of inhaled drugs. Thus, we used the human reperfusion model in a novel experimental context. The ventilated lung lobe offered the unique potential to administer the BDP formulation from a commercially available aerosol directly into the bronchus. The concentration of BDP and its metabolites could be then monitored by analyzing samples from the main venous vessel.

## Materials and Methods

### Chemicals, reagents and drug preparations

Beclomethasone dipropionate (BDP) pressurized metered dose inhalers (MDI) with hydrofluorocarbon (HFA) propellant (Sanasthmax^®^/Becloforte™ 250 μg/dose [Asche Chiesi GmbH, Hamburg, Germany] and Ventolair^®^/Qvar™ 100 μg/dose [3 M Medica, Neuss, Germany]) or chlorofluorocarbon (CFC) propellant (Sanasthmax^®^/Becloforte™) were obtained from a local pharmacy. BDP, beclomethasone-17-propionate (17-BMP), beclomethasone-21-propionate (21-BMP), beclomethasone (B) and fluticasone propionate (FP) were a generous gift from GlaxoSmithKline (Greenford, England). Diethylether (HPLC grade) was purchased from Fluka (Buchs, Switzerland) and acetonitrile (ACN, HPLC gradient grade) from Fisher Scientific (Schwerte, Germany). Water was obtained from a Millipore™ water purification unit. Bovine serum albumin (BSA), dextrane 70000, and N-(2-hydroxyethyl)piperazine-N'-2-ethanesulfonic acid (HEPES) were purchased from GERBU (Heidelberg, Germany), glucose monohydrate from Gruessing GmbH (Filsum, Germany), and stock solution containing 10000 IU/mL penicilline and 10000 μg/mL streptomycine in 0.9% NaCl from Biochrom AG (Berlin, Germany). All other chemicals were obtained from E. Merck (Darmstadt, Germany).

### Source and handling of human specimen for dialysis and scanning electron microscopy experiments

Human lung tissue specimen were obtained from patients with bronchial carcinomas who gave informed consent. Only cancer-free tissue was used for the experiments. None of the patients was treated with glucocorticoids for the last 4 weeks prior to surgery. Tissue samples were shock frozen in liquid nitrogen after resection and stored at -70°C until usage. To collect sufficient material for the experiments, tissue samples of three or more patients were pooled. Tissue was thawed and cut into small pieces. One part of the tissue pieces was homogenized in two parts of Krebs-Ringer-HEPES buffer (118 mM NaCl, 4.84 mM KCl, 1.2 mM KH_2_PO_4_, 2.43 mM MgSO_4 _× 6 H_2_O, 2.44 mM CaCl_2 _× 2H_2_O and 10 mM HEPES; pH = 7.4). Homogenization was performed under continuous cooling using an Ultraturrax (Janke & Kunkel, Staufen, Germany). Before starting the series of dialysis experiments the required amount of human lung homogenate was estimated and subsequently a sufficient amount was prepared and divided into aliquots. Since all aliquots descended from this preparation protein content and enzymatic activity of the homogenate was identical for each experiment.

Plasma samples were obtained from healthy volunteers who gave informed consent. Samples were either used immediately or were shock frozen in liquid nitrogen and stored at -70°C until usage.

Bronchial fluid was collected from patients undergoing bronchoscopy for diagnostic purposes after having obtained informed consent. Bronchial fluid was obtained through a sterile plastic catheter inserted into the biopsy channel of the bronchoscope (Olympus BF 1 T 30; München, Germany), wedged into a subsegment bronchus. Small bronchial fluid aliquots of four patients were collected and pooled. The specimen was frozen and stored at -70°C until usage.

### Patients and lung preparations for perfusion experiments

Six patients with a bronchial tumour undergoing standard thoracotomy, were included in the study. None of the patients was treated with glucocorticoids for the last 4 weeks prior to surgery. Only patients with tumours that were located peripherally within the lung lobe were included. Each patient signed a written informed consent before surgery, and a local Ethics Committee approved the use of resected human lungs for perfusion. Immediately after perfusion, the lung preparations were examined as usually by a pathologist.

### Dialysis experiments

**Figure 1:**
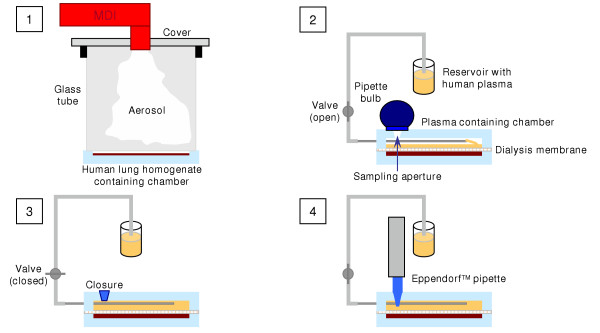
Schematic illustration of the dialysis experiments. [1] 500 μg BDP *ex-valve *is applied to human lung homogenate. [2] Dialysis membrane is placed on homogenate, the dialysis unit is closed by attaching the second chamber, and the second chamber is filled with human plasma. [3] Sampling aperture is closed and the dialysis unit is incubated by 37°C. [4] Sample of 500 μl is drawn and the volume is replaced with fresh plasma.

Dialysis was performed with a dialysis unit (designed by our working group) consisting of two individual tightly fitting Teflon chambers separated by a dialysis membrane (Figure 1). One chamber (inner diameter: 45 mm, internal depth: 3 mm) was prepared for lung tissue homogenate, the other chamber (inner diameter: 45 mm, internal depth: 6 mm) was supposed to be filled with human plasma. The chamber for plasma had two apertures for obtaining dialysis samples and addition of fresh plasma.

In initial experiments we evaluated the functionality and reliability of the experimental setting with respect to the convection, appropriate sample recovery and absence of trapped air. Therefore, the upper chamber was replaced by a chamber with a top made of acrylic glass instead of Teflon to monitor the processes within the unit. After filling the lower chamber with buffer the dialysis membrane was placed on this chamber and the dialysis unit was closed by attaching the second chamber. The complete and air bubble free filling of the chamber by smooth negative pressure was visually controlled. To check whether there was sufficient convection for a homogenous distribution of the analyzed compounds in the plasma and whether replacement of the sample volume was readily achieved, buffer in the reservoir was stained with a green dye. Then sampling was performed as described above. The appropriate moment for closing the valve was defined as the point of time where no visible flow of stained buffer into the chamber could be observed. It was also determined if any air bubbles gained access through the sampling aperture during sampling or after removing the pipette. Control experiments with the dye verified that the sample was not adulterated during the process of sampling and synchronous replacement of the sample volume with fresh solution. The degree of convection was controlled by following the distribution of the green dye into the clear buffer after sampling. A visually homogenous solution was obtained within a few minutes under experimental conditions. Accurate and reproducible sample volume was assured by weighing ten replicates of samples drawn under experimental conditions. Parameters for accuracy and reproducibility were within the specifications of the manufacture of the used pipettor.

For the dialysis experiments a 5 g aliquot of pre-warmed human lung homogenate (37°C) was filled into one chamber. 500 μg BDP *ex valve *was applied to the homogenate employing a dosing device (designed by our working group, Figure 1) that was composed of a fitting for the MDI, the tested MDI, and a glass tube to assure reproducible dosing conditions. Time in which the particle cloud was allowed to sediment was kept constant. The lung homogenate was stirred briefly for a fixed period of time. Subsequently, a dialysis membrane (Spectra/Por™ 6, MWCO 2000, Spectrum Laboratories, Rancho Dominguez, USA) was placed on the homogenate, the dialysis unit was closed by attaching the second chamber. The second chamber was filled with plasma of 37°C. Therefore, the valve connecting the dialysis unit to the plasma reservoir was opened and the dialysis unit was filled with plasma by producing a mild negative pressure using a pipette bulb. Afterwards the valve was closed again. The whole appliance was free of trapped air. The dialysis unit was incubated at 37°C for 6 hours (Incubator, Memmert, Schwabach, Germany). For sampling the aperture on the top of the unit was opened and samples were drawn using an Eppendorf™ pipette, pipette tips of which were tightly fitting to the sampling aperture. Samples of 0.5 mL were drawn. Therefore, the valve connecting the dialysis unit to the reservoir was opened synchronously to sample drawing. Replacement of the sample volume with fresh pre-warmed plasma occurred due to negative pressure produced by the pipette. The appropriate moment for closing the valve was determined in control experiments. Samples were stored at -20°C until further analysis. To determine the BDP dose that was actually applied to the homogenate by the respective aerosol one dialysis chamber was filled with 5 ml of Krebs-Ringer-HEPES buffer (pH = 7.4) containing 150 μg FP as internal standard instead of homogenate. Dosing was performed analogous to the dialysis experiments and the BDP concentration in buffer was analyzed.

### Analysis of drug concentrations in dialysis samples

Samples of the dialysis experiments were mixed with 50 μL internal standard solution (3 μg/mL FP in methanol) and extracted twice with 2 ml diethylether for 20 min using a roller mixer, followed by centrifugation at 3000 rpm (Labofuge II, Heraeus-Christ GmbH, Osterode am Harz, Germany) for 5 min. The combined organic phases were evaporated to dryness under a gentle stream of nitrogen at 25°C. The resulting residue was reconstituted in 0.2 mL methanol and analyzed by liquid chromatography using a Waters HPLC (Milford, USA) consisting of a 1525 binary pump, a 717plus autosampler and 2487 dual wavelength absorbance detector. Data collection and integration were accomplished using Breeze™ software version 3.30. Analysis was performed on a Symmetry C_18 _column (150 × 4.6 mm I.D., 5 μm particle size, Waters, USA). Typically, 20 μL of sample were injected, a flow rate of 1 mL/min was used, and detection wavelength was set to 254 nm. Mobile phase consisted of water containing 0.2 % (v/v) acetic acid (A) and acetonitrile (B). The gradient elution started at 62 % eluent A, decreasing nonlinearly to 53 % A by 22 min and finally decreasing linearly to 28 % eluent A by 18 min. The lower limit of quantification of the assay was 20 ng/mL for all glucocorticoids.

### Calculation of the fraction of applied dose determined in plasma

The amount of the parent compound BDP and its metabolites 17-BMP (active metabolite), 21-BMP and beclomethasone that were distributed from the lung tissue into plasma were determined and calculated as percentage of the applied dose. Amount A_i _of drug related to the parent compound found in whole plasma at i-th sample was calculated by the following equations:

(1) *V_P _*= *r*^2^• *π *• *h*



c_i _Concentration of the compound at the i-th sample [m/V]

h Inner height of the plasma containing chamber

MW Molecular weight of the compound

r Inner radius of the plasma containing chamber

V_P _Calculated volume of plasma in the dialysis unit

On basis of the calculated amount A_i _the release of the drug into plasma D_i _at the i-th sample can be expressed as percentage of applied dose using equation (3) or (4):



A_j-1 _Amount of drug related to the parent compound found in whole plasma at the (j-1)-th sample

D_applied _Actually applied dose to the human lung homogenate

### Human lung perfusion experiments

**Figure 2:**
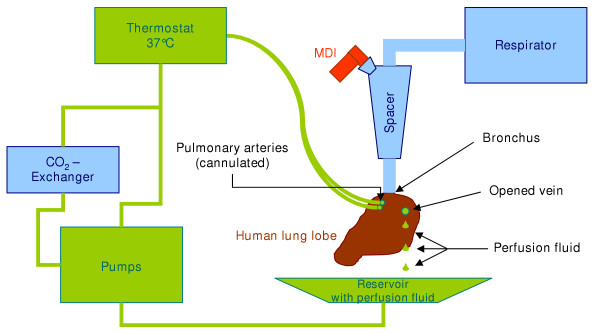
Schematic illustration of the experimental human lung reperfusion setting.

The lobe preparations were perfused extracorporally for about 60 min as described previously [14,15]. Immediately after lung resection, the pulmonary arteries were cannulated and the bronchus was connected to a bronchial tube. After the lung was rinsed through the arteries with 0.5 L of perfusion buffer (85 mM NaCl, 3.5 mM KCl, 2.5 mM CaCl_2 _× 2 H_2_O, 1.18 mM MgCl_2 _× 6 H_2_O, 2.5 mM KH_2_PO_4_, 20 mM NaHCO_3_, 5.5 mM glucose, 5 % bovine serum albumin and 2 % dextran; 100 μl of a stock solution containing 10,000 IU penicilline and 10,000 μg/mL streptomycine in 0.9% NaCl were added to 1 L perfusion buffer and pH was adjusted to 7.4 by addition of 10% NaHCO_3_), it was placed within the perfusion apparatus in a tempered water bath (37°C) and ventilated using a respirator (Engström Erica 2; Engström Elektromedizin GmbH, München, Germany) with air (Figure 2). The perfusion buffer was pumped from a reservoir through a heat exchanger, an oxigenator, and a bubble trap and was delivered through a valve into one to three segmental arteries. After leaving the opened vein, perfusate flowed back to the reservoir, which was held at 37°C.

During lung perfusion, pH, pO_2_, pCO_2_, K^+^, and Na^+ ^in the perfusate were monitored continuously with an Eschweiler System 2000-D03 (L. Eschweiler & Co., Kiel, Germany) and registered via a computer system. By addition of CO_2 _using a conventional oxigenator, perfusate pH was maintained within the physiological range of about 7.4. Lung preparations were weighed before and after perfusion to check for oedema formation during perfusion.

After ventilation and perfusion were established the system was equilibrated for 5 min, and a 5 mL sample (blank sample) was drawn before administration of the glucocorticoid. Dosing was performed using a glass spacer (constructed by our group) that was placed between the respirator and the ventilated lung tissue. During application the tidal volume was increased 1.5-fold in comparison to the volume during perfusion. The dose applied *exvalve *was 500 μg BDP for each MDI. Samples of 5 mL were drawn from the main venous output and the sample volume was replaced with fresh perfusion buffer. Samples were immediately frozen and transported on dry ice, and stored at -20°C until further analysis. In order to calculate the fraction of dose reaching the human lung lobe the spacer and all fittings were rinsed quantitatively and analyzed for BDP. The fraction of BDP that was determined in the spacer and connecting fittings was subtracted from the dose of 500 μg applied *ex valve*. The amount of the parent compound BDP and its metabolites 17-BMP (active metabolite), 21-BMP and beclomethasone that were distributed from the lung tissue into perfusion fluid were determined and calculated as percentage of the applied dose analogous to the dialysis experiments.

### Analysis of drug concentrations in perfusion samples

Perfusion samples of 1 mL were mixed with 25 μl internal standard solution (300 ng/mL FP in methanol). Glucocorticoids were extracted twice with 3 mL diethylether for 20 min using a roller mixer, followed by centrifugation at 3000 rpm (Labofuge II, Heraeus-Christ GmbH, Osterode am Harz, Germany) for 5 min. The organic layers were evaporated to dryness under a gentle stream of nitrogen at 25°C. The residue was reconstituted in 50 μl methanol and analyzed by HPLC – MS/MS using an Agilent 1100 HPLC system (Agilent Technologies, Waldbronn, Germany) consisting of a binary pump, a vacuum degasser, a temperature-controlled autosampler and a variable wavelength absorbance detector coupled with an Agilent LC/MSD Trap SL mass sensitive detector. An ESI interface was used in the positive ionization mode. Data collection and integration were accomplished using ChemStation-for-LC-3D™ and LC/MSD-Trap™ version 4.2 software. Analysis was performed on a Symmetry C_18 _column (150 × 4.6 mm I.D., 5 μm particle size, Waters, USA). The mobile phase was a mixture of water containing 0.1 % (v/v) formic acid (A) and acetonitrile (B), the flow rate was 0.6 mL/min. A gradient elution started at 50 % eluent B, increasing linearly to 65 % B by 8 min and then increasing linearly to 80 % B by 8 min. A total sample volume of 25 μL was injected. The mass spectrometer was operated in selective reaction monitoring observing the transitions from 465 m/z to 355 m/z, 501 m/z to 313 m/z, and 521 m/z to 411 m/z for 17-BMP, FP (internal standard) and BDP, respectively. The lower limit of quantification of the assay was 400 pg/mL for BDP and 17-BMP.

### Particle topology and dissolution of drug crystals in human bronchial fluid visualized by scanning electron microscopy (SEM)

Beclomethasone dipropionate (BDP) from different devices was either directly applied on regular glass microscopy slides or on microscopy slides with incubation slots of 15 × 2 mm (Karl Roth GmbH, Karlsruhe, Germany) containing human bronchial fluid. For application of BDP the glass microscopy slide was placed in a plastic spacer of about 900 cm^3^. The aerosol device was actuated and the glucocorticoid particles were allowed to alight on the glass slide. Number and distribution of BDP particles was visually controlled by light microscopy (ECLIPSE TS100, Nikon, Düsseldorf, Germany). After BDP application onto human bronchial fluid the glass microscopy slide was sealed with silicone paste and a cover slide and incubated at 37°C for one hour. Thereafter the cover slide was removed and the fluid was evaporated under a gentle stream of nitrogen. Subsequently the slide was carefully washed twice with 50 μL purified water to remove bronchial fluid proteins. The water was evaporated under a stream of nitrogen.

A Zeiss DSM 962 scanning electron microscope (Carl Zeiss, Oberkochen, Germany) was used to obtain the SEM photographs. The samples mounted on the glass microscopy slides were coated with palladium/coal for 3 min using a Baltec SCD 005 sputter-coater in an argon atmosphere (45 Pa and 50 mA).

## Results

### Lung tissue-plasma distribution of HFA-BDP determined in a dialysis model

The time course of distribution of two different HFA-BDP formulations from human lung tissue into human plasma was initially determined using a simple dialysis model. Therefore, equal doses of 500 μg BDP (2 × 250 μg Sanasthmax^®^/Becloforte™ and 5 × 100 μg Ventolair^®^/Qvar™) *ex valve *were applied to each 4.8 mL human lung tissue homogenate (Figure 1). The mean total dose that was actually deposited in this dialysis chamber was analyzed by HPLC and calculated to be 152.8 ± 15.4 μg for Sanasthmax^®^/Becloforte™ and 60.6 ± 1.5 μg for Ventolair^®^/Qvar™. Subsequently the distribution of BDP and its metabolites from lung tissue into the second chamber filled with 9.5 mL human plasma was monitored over 6 hours.

**Figure 3:**
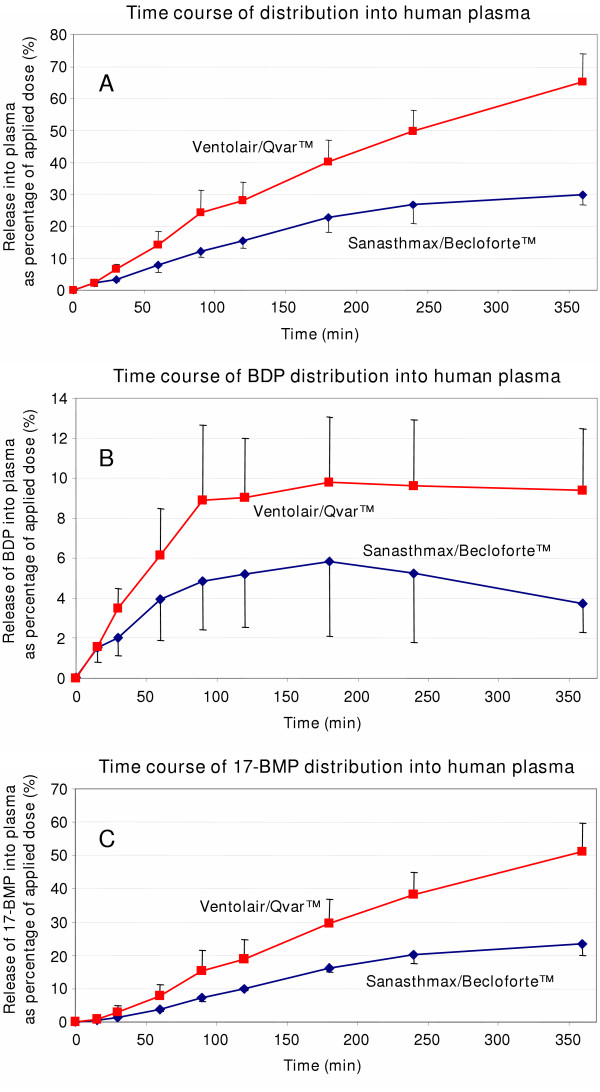
Time course of distribution of Sanasthmax^®^/Becloforte™ and Ventolair^®^/Qvar™ from human lung tissue into human plasma at 37°C as determined in dialysis experiments. Concentrations of [3 A] sum of beclomethasone dipropionate (BDP) and its metabolites, [3 B] BDP and [3 C] beclomethasone-17-monopropionate (17-BMP) were analyzed in plasma and expressed as percentage of the total dose applied to the lung tissue. Each data point represents the mean and mean deviation of the mean of three independent experiments.

The time course of HFA-BDP distribution in to the plasma chamber was different for the two formulations (Figure 3). After application of Ventolair^®^/Qvar™ about 14 % of the applied dose was delivered into plasma after one hour. After 6 hours about 65 % of BDP and metabolites were determined in the plasma compartment. In contrast, after one hour only about 8 % of the total dose of Sanasthmax^®^/Becloforte™ was distributed into plasma. After 6 hours 30 % of Sanasthmax^®^/Becloforte™ were found in plasma (Figure 3 A).

When the time course of delivery into plasma was regarded separately for BDP and 17-BMP, respectively, the clear differences between the two formulations were confirmed. After 30 min of incubation the plasma concentration of BDP delivered from Ventolair^®^/Qvar™ was twice as high as that of BDP delivered from Sanasthmax^®^/Becloforte™ (Figure 3 B). This difference became even more pronounced after 6 hours of incubation when the BDP concentration from Sanasthmax^®^/Becloforte™ decreased while it remained constant after application of BDP from Ventolair^®^/Qvar™. Up to 10 % of the applied dose of Ventolair^®^/Qvar™ were released unmetabolized as BDP into plasma while only about up to 6 % of the total dose Sanasthmax^®^/Becloforte™ were released unmetabolized.

The release of the active metabolite 17-BMP into plasma steadily increased during incubation (Figure 3 C). After 6 hours about 50 % of the total applied dose of Ventolair^®^/Qvar™, but only about 25 % of Sanasthmax^®^/Becloforte™ was distributed into plasma.

### Pulmonary absorption of HFA-BDP in a human lung perfusion model

A human lung perfusion model was employed to determine whether the differences of the distribution kinetics between the two BDP formulations were also present in a more physiological model. Lung lobes of cancer patients were extracorporally ventilated and reperfused in a closed system at 37°C directly after resection. BDP aerosols were applied via a glass spacer after increasing the respiration volume to the 1.5 fold of the basal volume. Again equal *ex valve *doses of 500 μg HFA-BDP were administered, 2 × 250 μg of Sanasthmax^®^/Becloforte™ and 5 × 100 μg of Ventolair^®^/Qvar™. After rinsing the glass spacer and all connecting tubes the fraction of BDP that adhered to those materials was analyzed after each experiment. By subtracting this amount from the nominal administered dose we calculated that mean doses of 343 ± 13.3 μg of Sanasthmax^®^/Becloforte™ and 392 ± 40.1 μg of Ventolair^®^/Qvar™ were deposited in the lung lobes.

**Figure 4:**
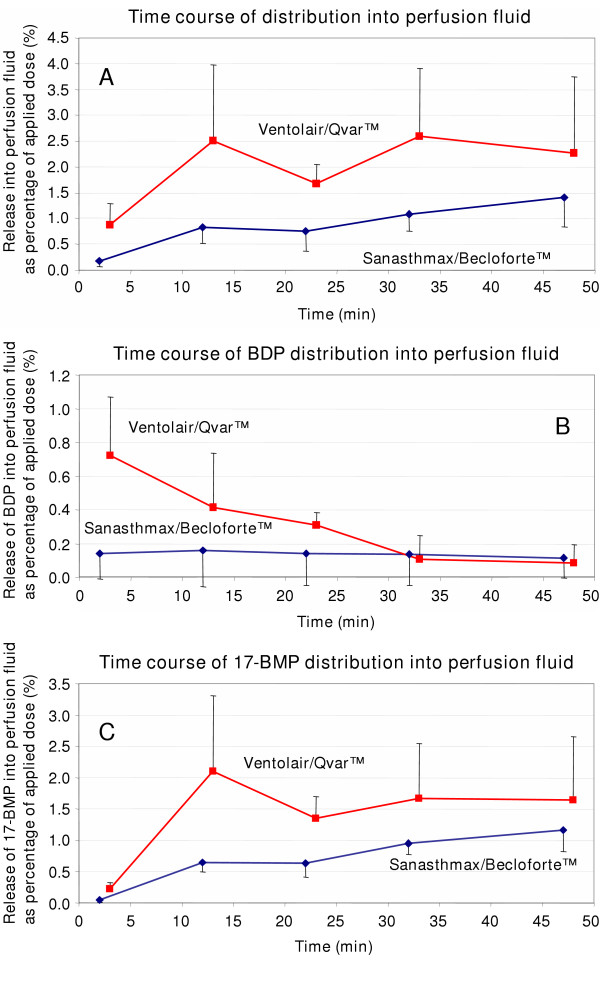
Time course of distribution of Sanasthmax^®^/Becloforte™ and Ventolair^®^/Qvar™ from a ventilated human lung preparation into perfusion fluid at 37°C. Samples were obtained form the main venous vessel of the lung lobe and the drug concentration was expressed as percentage of the total dose applied to the lung lobe. [4 A] Sum of beclomethasone dipropionate (BDP) and its metabolites, [4 B] BDP and [4 C] beclomethasone-17-monopropionate (17-BMP). Each data point represents the mean and mean deviation of the mean of three independent experiments.

Samples of the perfusion buffer were obtained directly from the main venous vessel of the lobe. Again the time course of HFA-BDP distribution into the perfusion fluid was different for the two formulations (Figure 4). The mean percentage of the applied dose that was delivered into the perfusion fluid after application of Ventolair^®^/Qvar™ was at all time points about twice a high as the distributed dose of Sanasthmax^®^/Becloforte™ (Figure 4 A). After application of Ventolair^®^/Qvar™ about 0.8 % of the BDP was immediately detectable in the perfusion fluid (Figure 4 B). This equals a mean concentration of 1.7 ng/mL in the perfusion fluid after only 3 min. Die BDP concentration then rapidly decreased over 30 min and then remained stable at a very low level. Though a very low percentage of BDP was also detectable in the perfusion fluid after application of Sanasthmax^®^/Becloforte™ it did not change over the incubation period. Only about 0.18 % of the total dose of BDP was continuously distributed into the perfusion buffer.

Analysis of 17-BMP concentrations after aerosol administration confirmed the differences in distribution observed between the two formulations. Over the whole incubation period about twice as high 17-BMP concentrations were observed after application of Ventolair^®^/Qvar™ compared with Sanasthmax^®^/Becloforte™ (Figure 4 C). While between 1.5 and 2 % of 17-BMP of the total applied dose from Ventolair^®^/Qvar™ were found, only between 0.5 and 1 % of 17-BMP were detected in the perfusion fluid after application of Sanasthmax^®^/Becloforte™. Interestingly, the active metabolite 17-BMP was already detectable in the perfusion fluid 2–3 min after application of both aerosols. As expected, the concentration of 17-BMP in the perfusion fluid gradually increased over the incubation time. This is consistent with a slow dissolution process of drug crystals.

### Particle topology and dissolution of drug crystals in human bronchial fluid visualized by scanning electron microscopy (SEM)

**Figure 5:**
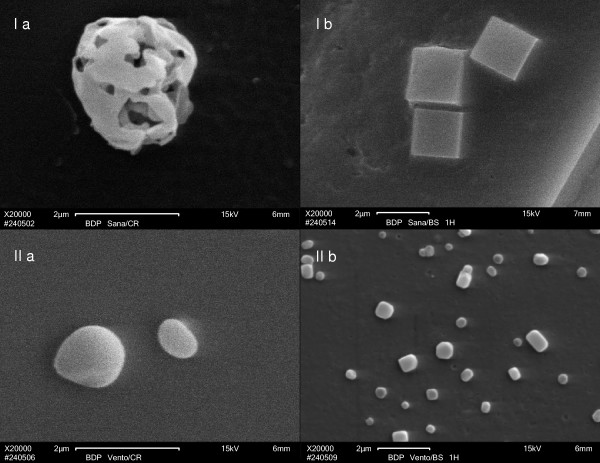
Scanning electron microscopy image of BDP particles either as delivered directly from the device on a glass slide [I a, II a] or in the stage of partial dissolution after incubation for one hour at 37°C with human bronchial fluid [I b, II b]. [I a] BDP particle as delivered from the Sanasthmax^®^/Becloforte™ formulation propelled by hydrofluoroalkane (HFA), [I b] HFA-BDP particles delivered from the Sanasthmax^®^/Becloforte™ formulation after incubation with human bronchial fluid at 37°C for one hour, [II a] HFA-BDP particles as delivered from the Ventolair^®^/Qvar™ formulation, [II b] HFA-BDP particles delivered from the Ventolair^®^/Qvar™ formulation after incubation with human bronchial fluid at 37°C for one hour.

Beclomethasone dipropionate (BDP) particles delivered by pressurized metered dose inhalers (MDI) with hydrofluorocarbon (HFA) propellant (Sanasthmax^®^/Becloforte™ and Ventolair^®^/Qvar™) were analyzed by SEM. To visualize the particle topology the devices were actuated and the particles were allowed to alight directly on regular glass microscopy slides. Representative HFA-BDP particles delivered by Sanasthmax^®^/Becloforte™ were about 2 μm (Figure 5, I a). BDP delivered by Sanasthmax^®^/Becloforte™ is solved in the HFA propellant. The particle size visualized by SEM is consistent with the published mass mean median aerodynamic diameter of about 2.6 μm [17]. These BDP particles showed a unique structure, they appeared round and highly porous. Obviously, a big surface area of the sponge-like form was generated by a rapid evaporation of the propellant.

To get an impression of the process of dissolution of these BDP particles in human bronchial fluid the Sanasthmax^®^/Becloforte™ device was actuated and the glucocorticoid particles were allowed to alight on microscopy slides with incubation slots. Each incubation slot accommodated about 40–50 individual BDP particles as controlled under a light microscope. The incubation slot was filled with 200 μL human bronchial fluid, sealed and incubated for one hour at 37°C. After this incubation time the SEM picture revealed that the form of the particles changed significantly. While the particles were still around 2 μm they now resembled solid cubic crystals (Figure 5, I b). Obviously, BDP particles re-crystallized after they came in contact with the bronchial fluid and formed crystals with a thermodynamically preferred smaller surface area.

BDP delivered by Ventolair^®^/Qvar™ is solved in the HFA propellant. BDP particles delivered by Ventolair^®^/Qvar™ are smaller than those delivered by the Sanasthmax^®^/Becloforte™ device. Representative particles shown (Figure 5, II a) are about 1 μm which is in agreement with the published mass mean median aerodynamic diameter of about 1.1 μm [18,19]. Again, these particles showed a typical structure, they appeared round and not porous, but droplet-like.

This BDP formulation was also incubated with human bronchial fluid for one hour at 37°C. The form of the particles changed to solid cubic crystals as seen with the other HFA-BDP formulation. However, most of the particles were now clearly smaller than 1 μm and the edges of the cubes appeared rounded. Obviously, the crystals were in a more advanced state of dissolution in bronchial fluid (Figure 5, II b).

**Figure 6:**
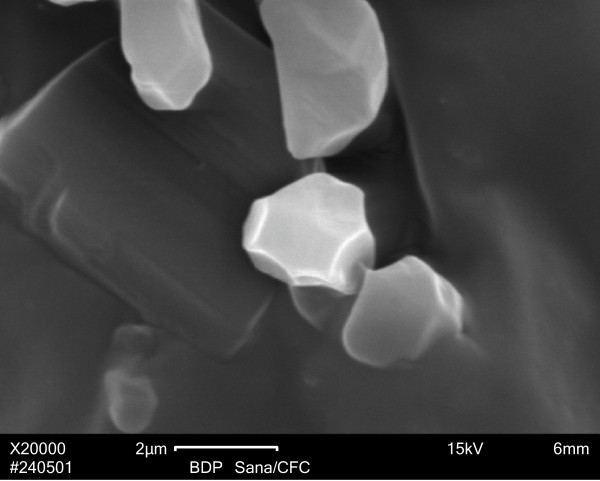
Scanning electron microscopy image of BDP particles as delivered from the Sanasthmax^®^/Becloforte™ formulation propelled by chlorofluorocarbon (CFC).

For comparison, we show a SEM image of BDP formulated as Sanasthmax^®^/Becloforte™ delivered from a MDI with CFC propellant and applied directly on a glass microscope slide (Figure 6). The particles were typically crystal-like, clearly bigger and less homogeneous than the particles delivered from any of the HFA driven aerosols. The size of these representative crystals is again consistent with the published mass mean median aerodynamic diameter of about 3.5 to 4.0 μm [18,19].

## Discussion

In the present study we successfully demonstrated that the intial distribution of beclomethasone dipropionate (BDP) from lung tissue into extrapulmonary circulation can be assessed employing a human lung perfusion model. Therefore, we applied BDP delivered by two different commercially available HFA-propelled aerosols (Sanasthmax^®^/Becloforte™ and Ventolair^®^/Qvar™) into a reperfused and ventilated lung lobe and measured the rate and extend of pulmonary absorption. We succeeded to explain kinetic differences we observed between the two aerosols with unique particle topology and divergent dissolution behaviour in human bronchial fluid.

Prolonged retention times in the therapeutic target tissue lung are a highly favourable characteristic of inhaled glucocorticoids and contribute to an improved therapeutic index. This principle was obviously taken into consideration for newly developed glucocorticoids for pulmonary application. All inhaled glucocorticoids of the latest generation reveal high lipophilicity and high tissue binding affinity. As additional mechanisms of tissue retention the formation of intracellular conjugates was reported for budesonide and ciclesonide's active metabolite [20,21].

Compound properties such as dissolution of drug crystals [22,23] and the glucocorticoids' tissue binding affinity [24] might be separately elucidated *in vitro. *The general differences determined between various glucocorticoids *in vitro *roughly agree with relation between tissue and plasma concentrations of these compounds at a certain time after inhalation in patients [9-11]. To elucidate the kinetics of pulmonary absorption plasma samples of patients or volunteers might be collected over a certain time interval after inhalation [12]. However, with this approach it cannot be excluded that the concentration of active drug being absorbed from the lungs is slightly underestimated due to partial hepatic metabolism. If glucocorticoids with a significant oral bioavailability are investigated gut absorption after swallowing has to be discerned from pulmonary absorption.

We evaluated novel approaches to elucidate pulmonary absorption of an inhaled glucocorticoid *in vitro*. Therefore, we compared BDP formulations delivered from two different commercially available pressurized metered dose inhaler devices. With both experimental settings, the dialysis experiments and the human lung perfusion model, we had precise information about the dose applied to the lung tissue. With both models were able to study the combined processes of drug dissolution, activation to the active metabolite beclomethasone-17-monopropionate (17-BMP) and the distribution of BDP and 17-BMP from lung tissue into plasma or perfusion fluid, respectively.

We used the newly developed dialysis model to monitor the kinetics of drug diffusion over six hours. During this time we observed constant drug transfer into plasma and continuous generation of the active BDP metabolite. The latter suggests that the non-specific esterases responsible for pro-drug activation were functional within the chosen time interval. We ventilated and reperfused the resected human lung lobe over about one hour to be as close as possible to physiological conditions and avoid problems such as oedema formation that might be associated with prolonged perfusion times. The time interval of one hour is consistent with the time to reach maximal plasma levels (t_max_) of BDP and its ester metabolites after inhalation of HFA-BDP in various clinical studies [25-28]. Since these peak plasma levels were observed between 0.34 [28] and 0.9 hours [25] the reperfusion time of one hour appeared acceptable for our experiments. In our human lung reperfusion model we observed a continuous generation of 17-BDP and rapid transfer of BDP and 17-BMP into the perfusion fluid. Though the absorption rates of BDP and 17-BMP were quantitatively higher in the dialysis model, they were qualitatively consistent in both models.

Unexpectedly, we observed differences between the two HFA aerosol formulations Sanasthmax^®^/Becloforte™ and Ventolair^®^/Qvar™ in both the dialysis and the lung reperfusion setting. The mean release of BDP and 17-BMP from lung tissue into plasma or perfusion fluid was about twice as high after application of Ventolair^®^/Qvar™ compared with Sanasthmax^®^/Becloforte™.

In contrast to the unanticipated results of our absorption experiments with two HFA formulations, differences in pulmonary absorption after administration of CFC-BDP compared to HFA-BDP would have been expected based on the results of clinical studies. After inhalation of HFA-propelled BDP higher lung deposition rates were achieved and higher plasma concentrations of 17-BMP were observed in adults [25,26] and children [28]. The rationale of a higher lung deposition and thus the need for lower doses of HFA-BDP in clinical settings is the difference in fine particle mass compared to the CFC-BDP [29]. The smaller particles delivered by HFA formulations result in a higher respirable fraction and improved deposition of particles especially in smaller airways [30]. The higher lung deposition of smaller drug particles correlates with a shorter the time to maximum plasma concentration (t_max_) of 17-BMP or beclomethasone esters, respectively, after inhalation of HFA-BDP compared to CFC-BDP [25,26]. The median t_max _of 17-BMP after inhalation of CFC-BDP was not significantly altered when activated charcoal was co-administered and thus gut absorption was prevented [17,31]. A plausible explanation for the delayed t_max _of 17-BMP after inhalation of CFC-BDP is the particle size as it should be expected that bigger drug crystals dissolve more slowly. HFA-BDP with its smaller particles exhibited a faster and more efficient systemic drug delivery [26].

Based on the comprehension of the interrelation of aerosol particle size and rate of pulmonary absorption we suspected that the different time courses of distribution into extrapulmonary compartments we observed were due to different particle sizes of HFA-BDP formulated as Sanasthmax^®^/Becloforte™ or Ventolair^®^/Qvar™, respectively. Scanning electron microscopic experiments illustratively confirmed this postulate. Particles of BDP delivered by Ventolair^®^/Qvar™ were significantly smaller and displayed faster dissolution in human bronchial fluid compared to particles delivered by Sanasthmax^®^/Becloforte™. Additionally, for the first time we provide evidence that BDP particles delivered by HFA-propelled aerosols are subjected to a re-crystallization process once they come in contact with the bronchial fluid. Obviously, the particles as delivered from the device dissolve rapidly due to their large surface area. Subsequently, locally oversaturated solutions tend to crystallize and crystals with a thermodynamically preferred smaller surface area are formed. It seems very likely that this might also occur in the life human lung since the volume of bronchial fluid is very limited. This re-crystallization of particles with high surface area to solid cubic particles with smaller surface area would be a highly desired effect as it slows down the further dissolution and therefore prolongs the pulmonary residence time.

The notion that BDP delivered by HFA-propelled inhalers initially rapidly dissolves and then locally re-crystallizes would also explain the detection of BDP in reperfusion fluid of the human lung preparation after only 2–3 min. It seems plausible that a certain amount of dissolved drug is instantly distributed into extrapulmonary compartments since the human lung has a high inner surface area and is the organ with the highest blood flow of about 15 L/min/kg [32]. The detection of BDP in the perfusion fluid is consistent with earlier studies reporting unchanged BDP in plasma samples of volunteers after inhalation of BDP [17,31]. Interestingly, an enhanced pulmonary absorption rate after inhalation of HFA-BDP compared to CFC-BDP was reported to correspond with an increased absorption of unchanged BDP [17,33]. In our human lung perfusion model we also detected higher levels of BDP in perfusion fluid after application of Ventolair^®^/Qvar™ with its smaller BDP particles. The fact that we also detected the active metabolite 17-BMP in the perfusion fluid of the extracorporally perfused and ventilated lung lobe after only 2–3 min allows the conclusion that the hydrolysis of BDP is an extremely rapid process. This is in excellent agreement with the results of earlier *in vitro *assays with human lung tissue [34]. Hence, the activation of the pro-drug BDP to 17-BMP is no rate-limiting step while the dissolution of drug crystals determines the rate of pulmonary absorption.

To summarize, we employed two different models to monitor the distribution kinetics of two HFA-propelled BDP aerosols from human lung tissue into extra-pulmonary compartments. With both the dialysis setting as well with the human lung perfusion model we detected significant differences between the two BDP formulations. The HFA-BDP formulated as Ventolair^®^/Qvar™ displayed a rapid release from lung tissue while Sanasthmax^®^/Becloforte™ exhibited a prolonged residence time in tissue. We succeeded to explain the observed differences in lung tissue residence time on the basis of the particle size and dissolution behaviour in human bronchial fluid. We conclude that though the ultrafine particles of Ventolair^®^/Qvar™ are beneficial for high lung deposition [30,35], they also yield an undesired more rapid systemic drug delivery. While the differences between Sanasthmax^®^/Becloforte™ and Ventolair^®^/Qvar™ were obvious in both the dialysis and lung perfusion experiments, the latter allowed to record time courses of pro-drug activation and distribution that were more consistent with results of comparable clinical trials. Though it is not clear whether the kinetic differences observed between Sanasthmax^®^/Becloforte™ and Ventolair^®^/Qvar™ might be of clinical relevance the detection of divergent absorption kinetics confirm the functionality and sensitivity of our experimental setting. Thus, the extracorporally perfused and ventilated human lung is a highly valuable physiological model to explore the comparative lung pharmacokinetics of inhaled drugs.
